# Advances in Dendritic-Cell-Based Vaccines against Respiratory Fungal Infections

**DOI:** 10.3390/vaccines12090981

**Published:** 2024-08-28

**Authors:** Nitish A. Kulkarni, Som G. Nanjappa

**Affiliations:** Department of Pathobiology, College of Veterinary Medicine, University of Illinois Urbana-Champaign, Urbana, IL 61802, USA

**Keywords:** dendritic cells, pulmonary fungal infections, DC-based vaccine, adaptive immune response

## Abstract

Ever since the discovery of dendritic cells by Ralph Steinman and Zanvil Cohn in 1973, it is increasingly evident that dendritic cells are integral for adaptive immune responses, and there is an undeniable focus on them for vaccines development. Fungal infections, often thought to be innocuous, are becoming significant threats due to an increased immunocompromised or immune-suppressed population and climate change. Further, the recent COVID-19 pandemic unraveled the wrath of fungal infections and devastating outcomes. Invasive fungal infections cause significant case fatality rates ranging from 20% to 90%. Regrettably, no licensed fungal vaccines exist, and there is an urgent need for preventive and therapeutic purposes. In this review, we discuss the ontogeny, subsets, tissue distribution, and functions of lung dendritic cells. In the latter part, we summarize and discuss the studies on the DC-based vaccines against pulmonary fungal infections. Finally, we highlight some emerging potential avenues that can be incorporated for DC-based vaccines against fungal infections.

## 1. Introduction

Fungal infections represent a significant global health problem causing approximately 3.8 million annual deaths, mainly in immunocompromised individuals [1]. This mortality rate surpasses that of malaria and is nearly comparable to the deaths caused by HIV/AIDS and tuberculosis [2]. The severe global concern is mostly driven by pulmonary mycoses caused by fungal species such as *Aspergillus*, *Cryptococcus*, *Coccidioides*, *Histoplasma*, *Blastomyces*, and *Paracoccidioides*. The infections caused by these species can be wide-ranging from innocuous, self-clearing to invasive infections leading to severe complications, especially in individuals with compromised immunity, such as those with HIV/AIDS, organ transplant recipients, patients with comorbidities like SARS-CoV2 infection, or patients undergoing chemotherapy, radiotherapy, immunotherapy, or receiving anti-inflammatory agents. According to an estimate, the fungal diseases posed a significant healthcare burden and workforce loss of an estimated 11.5 billion USD [3] and, according to the CDC, it could be as high as 48 billion USD [4]. The current antifungal drug arsenal is limited due to increased drug resistance, severe toxicity, and a high cost [5,6,7]. To overcome these challenges, new strategies for their prevention and treatment are being developed, such as vaccines, immunomodulation, and passive immunotherapy, which can be employed to protect vulnerable patients.

In recent years, the development of dendritic cell (DC)-based vaccines has emerged as a promising frontier in the fight against respiratory fungal infections. Dendritic cells are essential innate immune cells, renowned for their role as *professional* antigen-presenting cells (APCs). These ‘large stellate cells’ were discovered in 1973 by two scientists named Ralph Steinman and Zanvil Cohn, who named them “dendritic cells” due to their unique structure like multiple extended dendrites or pseudopodia-like cytoplasmic protrusions in their maturation stage [8]. DCs possess the unique abilities of unique abilities in the uptake, processing, and presentation of antigens to T cells, thus initiating and orchestrating a tailored adaptive immune response [9]. DCs also exhibit diversity specialization into distinct subsets, each contributing uniquely to immune function, in different tissues. The major pulmonary DC subsets include conventional DCs (CD103^+^ cDC1, and CD11b^+^ cDC2), pDCs, and inflammatory or monocyte-derived DCs (infDCs/moDCs) [10]. cDC1 and cDC2 are involved in the induction of CD8^+^ and CD4^+^ T-cell responses, respectively, mainly due to cDC1’s exquisite ability to cross-present exogenous antigens via MHC-I molecules. However, monocyte-derived dendritic cells (moDCs), under the influence of inflammation, can modulate the ongoing T-cell responses [11,12]. Thus, these unique abilities of dendritic cells to induce a robust and specific immune response make them attractive candidates for vaccine targeting or development. Moreover, the advancements in dendritic cell biology and vaccine technology have facilitated the development of novel strategies to optimize their antigen cargo loading, presentation, and functions. Techniques such as extracellular vesicle (EV)-based or nanoparticle-based delivery systems are being explored to enhance the efficacy and durability of dendritic-cell-based vaccines against fungal infections [13,14] by improving antigen uptake, processing, and presentation, and maximizing their ability to elicit robust immune responses.

Despite the promising advancements, various challenges persist in the translational aspect of DC-based vaccines. Challenges include the optimization of vaccine formulations, the standardization of manufacturing, and ensuring safety, stability, and efficacy in diverse groups of patients. Further, the high cost and scalability of these vaccines must be addressed for broad clinical implementation. Moreover, the variability in individual immune responses necessitates further research to identify biomarkers that can predict vaccine efficacy and tailor treatments to individual patients.

In this review, we describe the panoply of pulmonary DC subsets, their origin, development, and their function in anti-fungal immunity in preclinical model systems. We emphasize the strategies involved in DC-based fungal vaccines, highlight the recent DC-focused studies in various pulmonary fungal infection settings, and discuss the prospects of dendritic-cell-based vaccines for the management of respiratory fungal infections and improvement of the patient’s disease outcomes.

## 2. Pulmonary Dendritic Cells (DCs)

**Figure 1:**
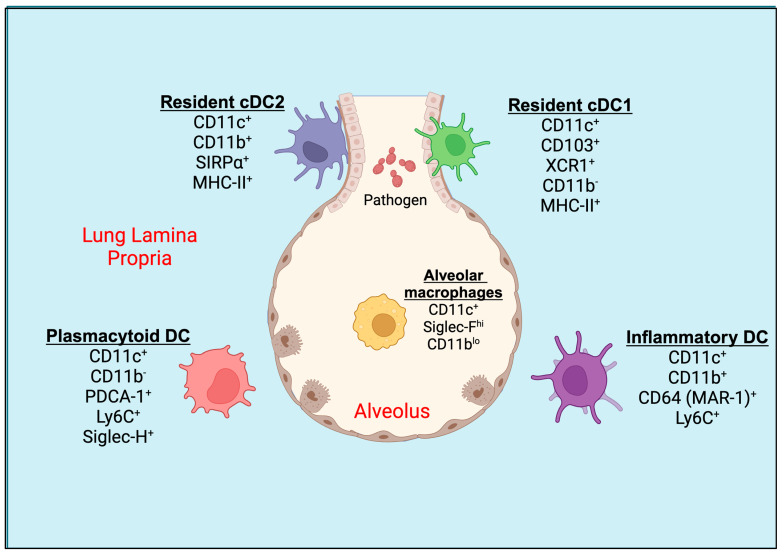
Pulmonary tissue DC subsets. Mucosal CD103^+^ resident cDC1s are located in the basolateral space of the epithelium and can extend their dendrites between epithelial cells directly into the lumen of the airway. CD11b^+^ resident cDC2 and plasmacytoid DCs (pDCs) are located underneath the basement membrane of the lung or in the lung parenchyma. During inflammatory conditions, an activated population of inflammatory DCs expressing CD11b^+^ and Ly6C^+^ are seen in the lung tissue. The alveolar macrophages (AMs) are present in alveolar space. DC, dendritic cell; cDC, conventional dendritic cell.

The lung is constantly exposed to particles during each breath and particle deposition in the airways occurs through impaction, sedimentation, interception, diffusion, and electrostatic precipitation largely dictated by the size [15]. Pulmonary DCs are lined along the airways (Figure 1), continuously sampling the environmental antigens, chemicals, and microbes including fungal spores and hyphal fragments as a part of surveillance [16,17]. With apt recognition, the antigens can be transported to the draining mediastinal lymph node or induce a specialized lymphoid structure called induced bronchoalveolar lymphoid tissue (iBALT) [18]. Thus, the airway is an important site and a target for intranasal vaccinations against inhaled fungi, and such vaccine platforms have shown promise in influenza model systems [19]. Here, we will describe the types and the role of pulmonary DCs.

The dendritic cells in the murine lungs are divided into conventional DCs (cDCs) and plasmacytoid DCs (pDCs; Figure 1). The lung cDCs express integrin CD11c at higher levels and are further classified as CD103^+^ DCs and CD11b^+^ DCs, also known as cDC1 and cDC2, respectively [20]. During the inflammation in the lungs, the monocytes can differentiate into DCs called monocyte-derived dendritic cells (moDCs) [10]. CD103^+^ cDC1s express MHC-II, Langerin (CD207), and high levels of the lymphotactin receptor XCR1, but low levels of CD11b [17,21,22]. The cDC1 creates an intricate network within the epithelial layer of the airways and extends elongated cellular projections into the spaces between basal epithelial cells for the sampling of the inhaled particles [17]. cDC1 engulfs soluble as well as apoptotic cell-associated antigens and migrates to the mediastinal lymph nodes during steady-state conditions and inflammation [23]. The cDC1 cells are well-known for their cross-presentation of antigens to cytotoxic T cells (CTLs) [24]. On the other hand, cDC2s express high levels of SIRPα (CD172α, a ligand for CD47), and intermediate levels of CX3CR1, MHC-II, and F4/80, but are mostly negative for CD103, XCR1, or CD207 [20,25]. They reside underneath the lamina propria or basement membrane of the lung [26]. CD11b^+^ cDC2 are specialized to present exogenous antigens via MHC-II to CD4^+^ T cells in mediastinal lymph nodes [12]. Both cDC1 and cDC2 migrate to mediastinal lymph nodes under steady-state conditions in the absence of inflammation to induce tolerance to inhaled antigens or particulates or allergens [27].

The airways also consist of pDCs that express PDCA-1 (CD317), Siglec-H, and B220, along with Ly6C, but not CD11b or SIRPα, which are distinct from moDCs [10]. Like cDC2, pDCs reside in the lung parenchyma, the site of gas exchange [16]. Following the exposure of foreign particles to the lungs, such as microbes (bacteria, fungi, parasites, and viruses), TLR ligands, environmental pollutants, or allergens, and the induction of inflammation, another subset of DC, CD11b^+^ moDC, is increased in the lung parenchyma and conducting airways [10]. The circulating monocytes, upon inflammatory stimuli, leave the blood vessel lumen within minutes to hours, cross the endothelial barrier, migrate towards the site of inflammation, and, with apt signals, differentiate into inflammatory DCs [28]. The surface markers of moDCs often overlap with CD11b^+^ cDC2 and express CD11b, SIRPα, F4/80, and MHC-II. It has been reported recently that the expression of CD64 (MAR-1 clone), MerTK, and Ly6C on moDCs helps differentiate from cDC2 in the inflamed lungs of mice [29,30,31]. Even though these subsets are more related to macrophages than DCs based on the MafB lineage, their superior ability of antigen presentation under ex vivo conditions makes them more of a DC phenotype [11,32,33,34]. Some studies have found that CD64^+^ moDCs account for ~25% of CD11c^+^CD11b^+^MHC-II^+^ lung DCs under steady-state conditions [10]. Further, the cytokine and chemokine production profiles of cDC2 and moDCs differ based on the nature of the pathogen [35]. In the murine lungs, alveolar macrophages also express higher levels of CD11c and often contaminate the DC gating population by flow cytometry. However, they can be distinguished from DCs by their higher autofluorescence, expression of higher levels of Siglec-F, and lower levels of CD11b, Ly6C, and MHC-II [36].

## 3. Origin and Development of Lung DCs

The origin and development of lung DCs are recently being defined [37]. The differentiation of progenitors occurs in the bone marrow and is a continuous process, necessitated by the constant need to replenish mature DCs [38]. The multipotent progenitors derived from hematopoietic stem cells undergo a series of phase transitions producing common lymphoid progenitors (CLPs) and common myeloid progenitors (CMPs) for lymphocytes and myeloid cells, respectively [39,40]. CMPs give rise to monocyte-dendritic cell progenitors (MDPs), which lack the potential to develop into granulocytes. The common dendritic cell precursors (CDPs) are identified as the first DC-restricted progenitors [41] and their development relies on the transcription factor FMS-like tyrosine kinase 3 ligand (FLT3L) and expresses ZBTB46 that is not essential for their development [42]. CDPs produce pre-DCs, which migrate to the peripheral organs and undergo local differentiation into mature cDCs within the tissue ambience [38]. The increased expression of interferon regulatory factor 8 (Irf8) is linked to the specification of plasmacytoid DCs (pDCs) and type 1 classical DCs (cDC1s). Here, Zbtb46 expression is crucial for cDC lineages, initially as pre-cDC1s and pre-cDC2s [40]. The transcription factors (TF) Basic Leucine Zipper ATF-Like Transcription Factor 3 (BATF3) and interferon regulatory factor (IRF8) dictate the lineage commitment of cDC1. Conversely, cDC2 development depends on the transcription factor IRF4 [43]. Fate-mapping studies reveal that almost all CD103^+^ cDC1 are derived from CDPs, whereas only 50% of CD11b^+^cDCs are accounted to be of CDP origin [44]. The other developmental pathways are still unknown. In contrast to cDCs, the complete development of pDCs occurs in the bone marrow before they migrate to the lungs, despite their closeness to cDC1 [45,46]. Interestingly, the ontogeny of pre-pDCs is from both CDP and CLPs, and, with their nature of high type-I cytokine producers, the debate exists to classify subsets of them as innate lymphocytes rather than DC [47,48]. pDC generation is mainly dependent on transcription factors like interferon regulatory factor 8 (IRF8), inhibitor of differentiation 2 (ID2), transcription factor 4 (TCF4), signal transducer and activator of transcription 3 (STAT3), and E26-transformation-specific (ETS) family member Spi-B [12,49,50]. In contrast to other subsets of dendritic cells, during infection and inflammation, bone-marrow-derived monocytes differentiate into monocyte-derived dendritic cells (MoDCs) or TNF- and iNOS-producing DCs (Tip-DCs). MoDCs, resembling classical monocytes, depend on macrophage-colony stimulating factor (M-CSF) for their development and CCR2 for their recruitment to inflamed lungs [51].

The studies employing bone marrow depletion techniques have elucidated that dendritic cells (DCs) migrating within the lungs undergo turnover every 2–3 days during steady-state conditions [52]. However, other studies suggested that 5–10% of the lung cDC proliferates at any given point [53]. Various factors, such as exposure to microbial antigens, tissue damage, and inflammatory signals, influence the turnover and replenishment rate of DCs in mucosal tissues. Parabiotic mouse experiments conducted in lung tissues have revealed a notable extended turnover period with CD11b^+^ DC and CD103^+^ DC exhibiting half-lives of 15 and 30 days, respectively [53]. Thus, the dynamics of different subsets of DCs and their homeostasis may influence the pathogenesis during infection.

## 4. Functions of DC against Respiratory Fungal Pathogens

While the role of DCs and their subsets are well-characterized during viral infection models, their studies during fungal infections are limited. Incontrovertibly, DCs are the single most common class of professional antigen-presenting cells that interconnect the innate immune system with adaptive immunity to pathogens including fungi [54]. It is conceivable that specific subsets of DC have distinct functions shaping the ongoing inflammation and T-cell responses. Further, the fungal pathogen can exist in different forms—the filamentous hyphal form, spore or conidia form, and single-cell yeast form—posing unique challenges to the dendritic cells for sensing, phagocytosis, killing, and antigen presentation [55,56].

**Figure 2:**
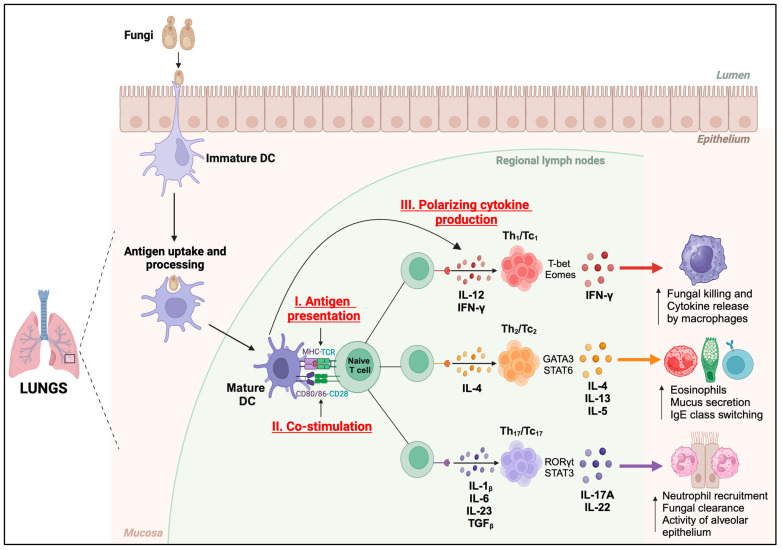
Functions of DCs. Immature dendritic cells (DCs) perform antigen uptake and processing in the mucosa of the lungs and present the antigenic peptides in MHC-I or MHC-II complexes to cognate TCRs on naïve T cells (Signal I) in regional lymph nodes. Fungal antigen recognition induces the maturation and activation of DCs, resulting in increased expression of co-stimulatory molecules, CD80 and CD86 (Signal II), which bind to CD28 on naïve T cells to initiate their activation. DCs secrete polarizing cytokines (Signal III) which induce the differentiation of T cells into lineage-specific subsets. Th_1_/Tc_1_ subset produces IFN-γ which enhances the fungal killing and cytokine release by macrophages. Th_2_/Tc_2_ subset secretes IL-4, IL-13, and IL-5, which increases the number of eosinophils, and mucus secretion, and promotes IgE class switching. Th_17_/Tc_17_ subset produces IL-17A and IL-22, which increases the neutrophil recruitment, fungal clearance, and alveolar epithelial cell functions.

The DCs activate and differentiate the T cells mainly by three signals (Figure 2); (i) antigen presentation, (ii) co-stimulation, and (iii) the production of lineage-defining cytokines. DCs recognize fungi through a diverse range of surface-bound and intracellular pattern recognition receptor (PRR) sensors [57]. The DC subsets cDC1 and cDC2 primarily process and present the fungal antigens via major histocompatibility complex (MHC) class I or MHC-II molecules to naïve CD8^+^ or CD4^+^ T cells, respectively. The migratory DCs (migDCs) engulf the antigen/yeast, migrate to the draining lymph nodes (dLN), and present the antigen to CD4^+^ and CD8^+^ T cells or transfer the antigens to cDCs [58]. On the other hand, the pDCs mainly produce type-I interferons and generally mediate immunity to viral infections [51]. moDCs mainly are involved during an ongoing inflammation and shape the ongoing adaptive immune responses. Notably, the process of recognition of fungi and the activation of DCs enhances the expression of the epitope-bound MHC molecules, co-stimulatory molecules (CD80/CD86), and polarizing cytokines essential for priming naïve T cells and their differentiation into multiple distinct subsets such as IFN-γ^+^ Th/Tc_1_, IL-4/13^+^ Th/Tc_2_, and IL-17^+^ Th/Tc_17_ [10,43]. Thus, the nature of DC activation determines the type of T-cell response and fungal immunity.

The functions of different DC subsets may depend on various factors, such as the site of infection/inoculation, the nature or phenotype of the fungal organisms, and the susceptibility of the host. For instance, a study in mice showed that Batf3^+^ cDC1 induces protective Th1 CD4^+^ T-cell responses and helps to clear cryptococcal infections [59] by upregulating T-cell recruitment, differentiation, and activation pathways. Contrastingly, conventional type 1 Langerin-expressing DCs (LangDC1) impaired immune responses against *Cryptococcus neoformans* in mice in the early stages of pulmonary infection [60]. This was associated with the downregulation of type-1 and type-17 cytokine production while enhancing the type-2 responses. In murine histoplasmosis, CD103^+^ cDCs were the main producers of type-I IFN in the lungs in a TLR7/9-dependent manner, and cause the induction of IFN-γ production by T cells and increased the host survivability [61]. CD11b^+^ DCs that recognized chitin during pulmonary cryptococcosis induced predominant Th2 cell responses, and their deletion using CD11c^cre^IRF4^fl/fl^ mice reduced the Th2 pathology [62]. Another murine study showed that CCR2 mediates CD11b^+^ DC (cDC2) recruitment to the lung during pulmonary cryptococcosis [63], which helps in mounting effective Th1 immune responses [63].

The various DC subsets cooperate with each other in inducing effective T-cell responses. For example, migratory DCs and moDCs shuttled the vaccine antigens to lymph-node resident DCs to induce robust vaccine-induced CD4^+^ T-cell responses that provided immunity against *Blastomyces dermatitidis* in mice [64]. Similarly, moDCs and neutrophils produce CXCL9 and CXCL10 through dectin-1, Card9-, and type-I and -III IFN-mediated signaling for the recruitment of pDCs and host defense in the mouse model of aspergillosis [65]. In another study, the FLT3L-dependent cDCs and CCR2-dependent moDCs presented the antigens and primed the T cells during mucosal candidiasis in mice [66]. In murine lung cryptococcal infection, IL-10 signaling reduced the numbers of moDC and the activation of CD11b^+^ type-2 DCs in an autocrine manner in persistent infection and the ablation of IL-10 promoted the host immunity by these subsets [67].

An important DC subset that increases after inflammation is monocyte-derived dendritic cells (moDCs)/inflammatory DCs. These TNF-expressing moDCs (Tip-DCs) effectively induced CD4^+^ T-cell responses and IL-17A-dependent airway neutrophilia and fungal killing, but the absence of dectin-1, MyD88, or TNF enhanced the IL-5 and impeded the fungal clearance in persistent aspergillosis in mice [68], suggesting TNF-producing moDC orchestrated neutrophil-dependent airway inflammation and immunity. Similarly, Notch ligands and receptors were upregulated phagocytes and Th1 cells during histoplasmosis, and the inhibition of Notch signaling led to an increased fungal burden in primary infection associated with the reduced differentiation and maturation of moDCs and elevated monocyte-derived alveolar macrophages polarized to M2 [69].

Interestingly, the conidial forms of yeasts can be phagocytosed effectively by DCs leading to a protective Th_1_ response by the expression of IL-12, but the hyphal or filamentous forms are not and may induce non-protective Th_2_ cells expressing IL-4 and regulatory cytokine IL-10 [70,71,72]. Human immature DCs seem to be activated upon exposure to *Aspergillus* germ tubes and expressed proinflammatory cytokines TNF and IL-12 in a dectin-1-dependent manner [73]. Interestingly, the murine moDCs seem to inhibit Th17 responses and promote Th1 responses, likely in a dectin-1-dependent manner, during aspergillosis [74]. The human DCs exposed to *Aspergillus* α-(1,3)-Glucan polarized naïve T cells to the regulatory type and the PD-L1 pathway facilitated this process while negatively regulating IFN-γ secretion [75]. The sensing of PRR ligands can dictate the outcome of T-cell responses. For example, the live but not the killed *Aspergillus* activated the dendritic cells mainly due to the differential beta-glucan display and activation of Th1 responses [76,77]. Despite the accumulating studies on the role of DCs during pulmonary fungal infections, distinct DC subsets’ functions directly contributing to immunity or by the activation and polarization of distinct cytokine-producing T cells or modulating other cells are not clear [78]. Further, only a few studies have directly evaluated the specific type of DCs in regulating the immunity to pulmonary fungal infections and we will discuss this where possible under specific DC-based fungal vaccines.

A relatively outlier DC subset, pDC, also has a role in antifungal immunity in a tissue-dependent manner [79]. A study showed that the deletion of pDCs increased the susceptibility of mice to invasive aspergillosis, and in vitro, fungal exposure to human pDCs enhanced the expression of TNF and type-I interferons and direct cytotoxicity [80]. Further, the blood-borne murine pDCs may crosstalk with the neutrophils to enhance NADPH oxidase activity and the *Aspergillus* conidial killing [65]. Despite the dectin-1 requirement for immunity to aspergillosis, dectin-2-mediated recognition by pDC was essential for TNF/IFN-α release, the formation of neutrophil extracellular traps, and antifungal activity in humans [81]. On the contrary, pDCs mediated the tolerance during lung infection with *Paracoccidioides brasiliensis* by inducing regulatory T cells [82]. *Paracoccidioides* infection augmented the number of pDCs’ immunoregulatory enzymes driving regulatory T-cell (Treg) expansion in an indoleamine 2,3-dioxygenase (IDO)-dependent manner [82]. Interestingly, dectin-1 activation in human myeloid DCs reduced the Th_2_ responses whereas dectin-1 activation in pDCs promoted the Th_2_ responses apparently due to their disparate surface expression of OX40L and reduced OX40L on myeloid DCs enhanced Th17 cell responses to curdlan treatment [83]. Thus, the pDCs have pattern recognition receptor (PRR)-specific, pathogen-specific anti- or pro-inflammatory immune responses to fungal pathogens.

Another notable function of DCs is “cross-presentation”; DCs process and present extracellular antigens via MHC-I molecules to activate CD8^+^ T cells [84,85,86]. DC-based cross-presentation is essential for initiating CD8^+^ T-cell responses against tumors and certain pathogens that do not directly infect antigen-presenting cells or use host translation machinery. The DC cross-presentation enables the immune system to detect and respond to a wider range of antigens than would be possible through direct presentation alone. Despite cDC1 being known to be potent cross-presenting cells, recent studies showed that cDC2, moDCs, and pDCs (in humans) also have a cross-presentation ability [12,54]. Most of these studies deciphered the cross-presentation ability of DC subsets in viral or tumor model systems, and their role during pulmonary fungal infections is scarce. In an in vitro study, the bone-marrow-derived DCs (BMDCs) engulf and cross-present *Histoplasma capsulatum* antigens either from live or killed yeast to cytotoxic CD8^+^ T cells or via the uptake of live or killed apoptotic macrophages that harbor *Histoplasma* spp. [87]. In hematopoietic transplanted patients, the TLR3 on CCR7^+^ migratory DCs enhances the memory CD8^+^ T-cell recall responses to *A. fumigatus* by detecting fungal RNA and promoting cross-presentation via MHC-I [88]. Despite the overwhelming data on cDC1 (CD8^+^ DC) for cross-presentation, a study showed that splenic cDC2 (CD4^+^ DC) could cross-present and activate CD8^+^ T cells using an OVA-expressing strain of yeast *Saccharomyces cerevisiae* [89]. Although these studies show that resident DCs can acquire and present fungal antigens, further studies are needed to unravel the subset of DCs involved in in vivo cross-presentation.

## 5. Dendritic-Cell-Based Experimental Fungal Vaccines

The DCs are the most principal innate cells to connect innate and adaptive immune systems mainly by the phagocytosis of fungal pathogens, antigen processing and presentation, co-stimulation, and secretion of polarizing cytokines for the activation, differentiation, and expansion of T cells (Figure 2) [17]. DC-based vaccines have shown promise in cancer immunotherapy and the treatment of infectious diseases [90]. Some of these vaccines have undergone testing in phase I, II, and III clinical trials for various cancers, including myeloma, melanoma, acute myelogenous leukemia (AML), ovarian cancer, and HNSCC [91,92,93,94,95]. Consequently, Sipuleucel-T (Provenge; Dendreon Corp, Seattle, WA, USA) became the inaugural autologous DC-based vaccine approved for treating specific cases of advanced prostate cancer [96]. Thus, the DC-based vaccine approach is gaining significance, and various strategies are being explored as a rational design for vaccines against pulmonary fungal infections, especially in immunocompromised hosts. There is insurmountable evidence that the T cells are instrumental in immunity to fungal infections, including by inducing an apt B cell response, and, not surprisingly, many DC-based vaccines are tailored to elicit T-cell responses.

Recent studies have examined several approaches to target DCs in the development of antifungal vaccines. One of the strategies involves pulsing DCs with fungal antigens through various methods, such as exposure to the fungal proteins, peptides, and cell wall extracts, or introducing fungal nucleic acids (DNA or RNA) [97]. This approach enables the utilization of fungal ligands like β-glucans, zymosans, and mannose, which can enhance effective antigen uptake by DCs and specific PRRs known as C-type lectin receptors (CLRs) signaling. This apt fungal recognition by PRRs activates DCs, allowing them to present antigens to T cells and initiate an adaptive immune response. Additionally, DCs are the cells that express higher levels of co-stimulatory molecules (CD80 and CD86) and cytokines (IL-1β, IL-6, IL-12, IL-23, and IL-4) which help in the priming and subsequent differentiation of T cells into various effector subsets (Th_1_, Th_2_, or Th_17_), and antibody responses following fungal vaccination [43,57].

Another approach involves targeting DCs for directed activation, the induction of inflammation, and the augmenting of T-cell responses. For example, employing nanoparticles that specifically target receptor DC-SIGN or DEC205 on dendritic cells (DCs) where nanoparticles delivered the PRR ligand and antigens initiating robust T-cell responses [98,99]. DCs are efficient in the endocytosis of mannosylated proteins in fungal pathogens via CLRs. For instance, the model antigen OVA upon mannosylation promoted its uptake by DCs through CLR family members DC-SIGN and CD206, leading to enhanced immunogenicity [99]. Similarly, DCs efficiently engulf antigen-loaded β-glucan particles mediated by the dectin-1 pathway [100,101]. This targeted strategy for DCs could potentially facilitate the loading of fungal antigens into DCs, which is essential for triggering effective cellular immune responses against fungal infections. Here, we have discussed the DC-based vaccine strategies that are utilized against a few pulmonary fungal infections, and some examples are listed in Table 1.

### 5.1. Aspergillus *spp.*

*Aspergillus* is a genus of globally ubiquitous filamentous fungi found in various environments including soil and decaying vegetation. Despite the abundance and frequent inhalation of *Aspergillus* spores (conidia), it rarely causes infection or immune diseases in healthy hosts [102,103]. However, in immunocompromised hosts and patients with pre-existing pulmonary infections, the spores can germinate into invasive hyphae, resulting in a variety of acute to chronic diseases [104,105]. The most common species of *Aspergillus* associated with invasive infections in humans include *A. fumigatus*, along with *A. flavus*, *A. niger*, and *A. terreus*, especially in patients receiving chemotherapy, stem cell, and solid organ transplants, and patients experiencing complications with COVID-19. Around 250,000 cases of invasive aspergillosis are reported annually worldwide and are associated with significant mortality rates [74,106]. Moreover, the sensitization to allergens from *Aspergillus* spp. leads to allergic diseases, and allergic bronchopulmonary aspergillosis contributes to ~5 million cases [106,107]. The current antifungal medications for aspergillosis have limited efficacy and may result in drug resistance. Thus, it is essential to develop therapeutic vaccines to control allergic responses and provide protection against these opportunistic fungi in immunocompromised individuals.

Dendritic-cell-based vaccines have emerged as a promising approach for treating aspergillosis. DC-based vaccines aim to harness the antigen-presenting capabilities of dendritic cells to stimulate a specific immune response to *Aspergillus* antigens. The experimental vaccines in mice have shown protection given by crude and genetically engineered *Aspergillus* antigens in a Th_1_ cell-dependent manner enhanced by the addition of an adjuvant, CpG oligodeoxynucleotides (CpG ODN) [108]. DCs undergo remarkable functional plasticity in response to the conidia and hyphae of *A. fumigatus* and provide in vivo antifungal immunity upon activation with fungal RNA or live fungi [56,109]. The protection to aspergillosis was mediated by DC-induced Th_1_ responses in a mouse model of allogeneic hematopoietic transplantation. Similar findings were observed in studies using recombinant *Aspergillus* proteins (such as Asp f 16) and CpG ODN as adjuvants [56]. Another study used a vaccine strategy involving the transduction of DCs with an adenovirus vector encoding the cDNA of IL-12 and primed with heat-inactivated *A. fumigatus* (HAF). The adoptive transfer of these genetically modified DCs into syngeneic mice stimulated antigen-specific Th_1_ responses, reduced the fungal burden, and improved survivability, suggesting the potential use of antigen-pulsed DCs and IL-12 gene therapy to treat invasive aspergillosis [110]. In some of these studies, the specificity of the adaptive immune response elicited by DCs pulsed with *Aspergillus* antigens may not be specific, which may reduce the effectiveness of these vaccines in providing targeted protection against aspergillosis. Nonetheless, this small clinical trial has shown promising results using DCs pulsed with antigens of *A. fumigatus* [111]. In this trial, patients who received DC-based therapy demonstrated protection against opportunistic infections following haploidentical transplants, suggesting that DC-based vaccines could be a viable option in preventing infections in high-risk individuals. Despite these positive outcomes, the high cost associated with DC-based vaccine therapies remains a significant barrier to widespread use. Further research is needed to optimize the efficacy and cost-effectiveness of these vaccines for wider implementations in clinical settings.

### 5.2. Coccidiodes *spp.*

*Coccidiodes* spp. especially *C. posadasii* and *C. immitis* are highly virulent, dimorphic fungi causing coccidioidomycosis or Valley Fever or San Joaquin Valley fever in the southwestern United States, northern Mexico, and regions of Central and South America [112]. The coccidioidomycosis has re-emerged as a major threat to human health due to climate change and newer geographical spread. In 2019, approximately 20,003 cases were reported to the Centers for Disease Control and Prevention (CDC) in the United States [113]. Patients’ travel history to the endemic areas is the primary cause of the disease reported worldwide [114,115,116]. Dissemination of endospores causes severe pulmonary infections or more widespread diseases affecting organs like the bones, skin, central nervous system, or other organs. The host’s susceptibility to coccidioidomycosis depends on age, racial or genetic predispositions, pregnancy, and immune status [117], and immunocompromised hosts (with AIDS), pregnant women, and patients with dysfunctional T cells are prone to developing severe disseminated forms of Valley Fever [118,119]. Many healthy individuals recover upon exposure to *Coccidiodes* arthroconidia, but around 5–10% develop active disease [113]. The current therapeutics against coccidioidomycosis are limited, expensive, and ineffective due to relapse or reactivation, and require prolonged use. Hence, there is a need for an effective vaccine or immunotherapy to treat Valley fever.

The earlier study on a DC-based vaccine against *Coccidioides* spp. in a mouse model utilized the strategy of transfecting the BMDCs with a plasmid DNA encoding for *Coccidioides*-Ag2/proline-rich antigen (PRA) [120]. A significant number of intranasally administered *Coccidioides*-Ag2/PRA-cDNA-transfected DCs (Ag2-DC) are retained in the mucosal organs, such as the lungs and gut [112,120], and stimulate the protective IFN-γ responses. A similar intranasal Ag2-DC immunization of highly susceptible BALB/c mice reduced the fungal burden, significantly increased the IFN- γ expression, and alleviated lung pathology. Further, this prophylactic Ag2-DC immunization increased the levels of serum IgG and its isotypes [121]. The indicators of lung injury (protein, lactate, and albumin) were normal, suggesting the DC vaccine itself did not cause any vascular leakage or tissue injury. Further studies showed that the DC vaccine provided IFN-γ-mediated vaccine immunity and protection against coccidioidomycosis in mouse models [122,123]. These DC-immunization strategies showed the feasibility of a therapeutic vaccine.

### 5.3. Paracoccidioides *spp.*

Paracoccidioidomycosis (PCM) is a systemic granulomatous infection caused by the *Paracoccidioides* spp. *(P. brasiliensis* and *P. lutzii)* of thermo-dimorphic fungi, which are endemic to Latin America (Mexico to Argentina), mainly affecting workers with significant soil exposures. The high incidence of PCM is reported in Brazil, where around 51% of the confirmed deaths due to systemic mycoses were caused by PCM from 1996 to 2006 [124,125]. Upon the inhalation of fungal propagules, they undergo differentiation into the infective yeast form in the lung, resulting in the development of characteristic granulomatous lesions through the activation of Th_1_ responses [126]. In immunocompromised individuals, the granulomatous lesions contain abundant viable fungi that have the potential to disseminate to almost all organs and tissues. Th_1_/Th_17_ responses provide protection against PCM, while Th_2_ responses and regulatory T cells (Tregs) lead to a severe form of the disease [127]. Interestingly, PCM vaccines aiming to reduce only the fungal burden are insufficient to lower the morbidity and mortality, and a well-balanced cytokine response remains essential [127,128].

Dendritic cells are shown to be important for initiating the adaptive immune responses for the control of *Paracoccidioides* spp. infections [126,129,130]. The peptide-based vaccine strategy involves utilizing the P10 peptide, an immunodominant antigenic region of the glycoprotein 43 (gp43) of the fungus *P. brasiliensis,* containing a sequence of 15 amino-acid epitopes specific for CD4^+^ T cells. The P10 antigen was used to test the adjuvant-like effects of DC presentation to enhance the resolution of PCM [113]. In experimental mouse models, BMDCs pulsed with the P10 antigen showed both therapeutic and preventive effects against PCM [131]. Upon the subcutaneous immunization of P10-primed DCs and the subsequent challenge with a virulent strain induced a strong Th_1_ response with an increased production of IFN-γ and IL-12, while reducing the IL-10 and IL-4 levels correlated with a reduction in the number of granulomas, the alleviation of lung damage, and a reduction in the fungal burden, suggesting its potential use as a therapeutic and prophylactic [131]. A study with a similar strategy but using monocyte-differentiated dendritic cells (moDCs) harvested from infected mice were pulsed with P-10 and used as a therapeutic vaccine, giving protection rivaling BMDC-P10-pulsed vaccination [132]. Perhaps, in humans, monocyte-derived DCs could be employed. In vitro experiments also demonstrated that the DC-based approaches activate and upregulate MHC-II, CD80, and CD86 expression on DCs and induce the proliferation of CD4^+^ and CD8^+^ T cells.

Considering the apprehension for PCM treatment in immunocompromised patients, the P10-pulsed DC vaccination has been investigated in BALB/c mice injected with dexamethasone to mimic the immunocompromised state. DC-vaccination effectively induces Th_1_-specific and protective immune responses against *P. brasiliensis*, the causative agent of a severe form of PCM [133]. Furthermore, a combination of antifungal drugs with a P10-primed DC therapeutic vaccine has a synergistic effect in reducing the fungal burden in immunosuppressed mice with PCM. These studies highlight a range of vaccine candidates for *Paracoccidioides,* with ongoing efforts to develop a P10-based adjuvant vaccine for clinical trials [127,131,132,133].

### 5.4. Cryptococcus *spp.*

Cryptococcosis is a highly virulent invasive fungal infection mainly caused by *Cryptococcus* spp. (*C. neoformans* and *C. gattii*). While *C. neoformans* infection primarily occurs in immunocompromised individuals, *C. gattii* can cause infection in apparently healthy individuals. In 1999, *C. gattii* infection emerged as an infectious disease in Vancouver Island, Canada, and surrounding regions, resulting in deaths among healthy individuals, with a mortality rate of around 8–20% [134,135]. The infection typically occurs through inhalation of the spores, leading to a primary lung infection. While many individuals may remain asymptomatic, the fungus can disseminate from the lungs to other parts of the body, mostly the central nervous system (CNS), causing conditions like meningitis. Patients with compromised T-cell immunity such as HIV/AIDS are highly susceptible to cryptococcosis. In 2020, around 152,000 cryptococcal meningitis cases were reported worldwide, resulting in ~112,000 deaths [136,137], with a case fatality rate of ~74%. Cryptococcosis accounts for 19% of all AIDS-related mortality. The increased global prevalence of cryptococcosis in immunodeficient hosts necessitates the development of new therapeutic avenues, including preventive vaccines.

The whole-cell-based DC-vaccine approach has garnered interest for protection against cryptococcosis. The study utilized the pulsing of BMDCs with the heat-killed acapsular *C. gattii* (∆cap60) strain of yeast, where the capsule synthesis-related gene cap60 was disrupted using a reverse genetic approach, and pulsed DCs were adoptively transferred intravenously into the mice twice before pulmonary infection with the highly virulent *C. gattii* strain R265 [138]. This vaccine strategy provided good immunity with a ~8900-fold decrease in the fungal burden, cross-strain protection, a significant reduction in lung pathology, and improved survival rates. The vaccine immunity increased the number of multinucleated giant cells (MGCs) that are involved in phagocytosis and enhanced the production of cytokines such as IL-17A, TNF, and IFN-γ, but reduced protection in mice lacking IFN-γ [138]. Concomitantly, pulsed DCs were required to establish immune memory against cryptococcus infections, called “trained immunity” [139,140] involving epigenetic modifications in the DCs. Several experimental vaccines have elucidated the significance of vaccination leading to protective immunity against cryptococcosis [141,142]. The ex vivo incubation of DCs with *C. neoformans* or immunizing with H99γ resulted in an increased production of IFN-γ and pro-inflammatory cytokines. Further, the responses showed a memory-like DC response, associated with epigenetic modification, which may aid in providing protective immunity to immunosuppressed hosts [140,143]. A recent study showed that the *C. gattii* Cap60∆ BMDCs preferentially migrated to the lungs, recruited recipients’ DCs and promoted long-lived tissue-resident memory Th_17_ cells protective against highly virulent *C. gattii*. The lung-resident memory Th_17_ cells activated neutrophils and bolstered granuloma formation, providing immunity to pulmonary cryptococcosis [144]. Overall, these findings suggest that systemic DC vaccines have the potential to induce protective immune memory against cryptococcal infections, particularly in the lungs; however, their effectiveness in humans needs to be studied further.

**Table 1:** DC-based fungal vaccines against pulmonary fungal infections.

Fungi	Vaccine Type	Methodology	Major Outcomes	References
*Aspergillus* spp.	RNA or live fungi/Crude antigen or subunit vaccine	Murine and human DC-pulsed (RNA complexed with DOTAP)	Enhanced DC’s MHC-II and co-stimulatory molecules expression, and increased Th_1_/Th_2_ response	[108]
	Heat-killed fungi/Crude antigen vaccine	Human DC-pulsed	In vivo protective antigen-specific Th_1_ response (high IFN-γ and IL-10 production)	[109]
	Heat-inactivated fungi/viral transduction	Murine DC-pulsed and IL-12 gene therapy	Increased Th_1_ responses, improved survivability, and reduced fungal burden	[110]
*Coccidioides* spp.	Ag2/Subunit vaccine (Ag2/PRA-cDNA transfected DC)	Murine transfected DCs	Reduced fungal burden, tissue injury in vaccinated mice, enhanced IgG levels, and increased IFN-γ, IL-4, and IL-17 production	[120,121,122,123]
*Paracoccidioides* spp.	Peptide vaccine (P10) P10 primary DC P10 primary monocyte derived-DC	P10-primed murine DCs	Reduced fungal burden in both immunocompetent and immunosuppressed mice, protection against intratracheal challenge, protective Th_1_ responses, activation and upregulation of MHC-II, CD80, and CD86 on the DCs, and induction of CD4^+^ and CD8^+^ T-cell proliferation.	[131,132,133]
*Cryptococcus* spp.	Heat-killed *Cryptococcus gattii* mutant ∆cap60	Murine DC-pulsed	Protection and stimulation of tissue-resident memory Th_17_ cells in the lungs	[138,144]
	Live or heat-killed *Cryptococcus neoformans* mutant	-	Protective Th_1_-type adaptive immune response Induction of trained immunity of DCs	[140]

DC: dendritic cell; DOTAP: cationic lipid N-[1-(2,3-dioleoyloxypropyl]-N, N, N,-trimethylammonium methyl sulfate; Ag2/PRA: protein termed antigen 2/proline-rich antigen.

## 6. Future Perspectives of Fungal DC Vaccines

Despite the several limitations of DC-based vaccines, new emerging techniques of inexpensive and easy culturing of monocyte-derived DCs from PBMCs in humans may bolster therapeutic vaccines in conjunction with antifungals. With traditional DC-based fungal vaccine candidates and strategies, new platforms and techniques are evolving to propel fungal vaccine research. The fungal extracellular vesicles (EVs) have great potential as a fungal vaccine candidate. EVs are lipid bilayer nanoparticles secreted by fungi, that can carry cargo of proteins, enzymes, lipids, nucleic acids, and carbohydrates [14]. For example, EVs from *C. neoformans* carry immunogenic proteins, like mannoprotein MP88, Vep proteins, and chitin deacetylase Cda family proteins—all of which have been tested as potential vaccine candidates [145]. In recent animal studies, EVs isolated from *Paracoccidioides* and *Candida* showed immunogenicity and vaccine immunity [14,146]. Similarly, the mice immunized with EVs derived from *Cryptococcus* acapsular cap59 mutants secreted antibodies against EV proteins and provided a strong immunity to the wild-type H99 challenge [145]. The possible advantage of an EV-based vaccine is its combination of various natural antigens which induce strong immunity. Further, EVs offer stable structural conditions for protein components, allowing them to circulate in bodily fluids and effectively sensed and taken up by antigen-presenting cells [147]. Some of the challenges with EVs are the heterogeneity of the contents, scaled-up production, and toxicity or immunosuppression of some components. Thus, EV-based antigen delivery may offer a novel approach to DC-based vaccines.

The nanoparticle-mediated targeting of DCs can potentially enhance the fungal vaccine efficacy due to their sub-micron size and specific targeting of DCs [13]. Nanoparticles can be composed of various materials such as fungal cell surface carbohydrates, metallic nanoparticles, and lipid-based particles, and serve as effective carriers for fungal antigens. Targeting dendritic cells (DCs) with nanoparticles allows for the precise delivery of specific antigens combined with an adjuvant and a DC-receptor-targeting (DEC-205) molecule [27]. Several studies depicted the effectiveness of using nanoparticles coated with DEC-205 F(ab’)2 fragment, containing TLRs such as TLR3, TLR7, and TLR8 ligands, and OVA antigen [98]. This combination strategy is designed to effectively target DEC-205^+^ DC and deliver both the antigens and adjuvants. This technique enhances the immunogenicity, increases the antigen delivery potency, and reduces the overall toxicity. Overall, the nanoparticle-based DC vaccines could be utilized against fungal infections to induce effective immune responses with limited toxicity.

The recent COVID-19 pandemic and the development of mRNA-based vaccines have accelerated the adoption of this technology, providing promise for the development of mRNA-based vaccines against other pathogens. RNA-based vaccines offer the benefits of a straightforward, rapid, versatile, scalable, modifiable, and cost-effective manufacturing process, as only the encoded RNA sequence needs to be modified. Further, mRNA is non-infectious, making it relatively safe to use in immunosuppressed hosts [148]. Additionally, it can be modified to self-replicate within the host, enhancing its ability to stimulate a robust immune response necessary for generating immunological memory [149]. Through the integration of cutting-edge vaccine technologies and the utilization of established mRNA vaccine platforms, one can accelerate the identification of novel and immunogenic fungal vaccine candidates, promising a potential breakthrough in fungal vaccine development in the near future. In the next decade, future research will explore integrating various methods of innovation for the development of vaccines against fungal infections. Strategies like DNA vaccines, subunit vaccines, attenuated vaccines, and advancements in adjuvant optimization should guide us to advance into new phases of innovative effective vaccine development.

## 7. Conclusions

In this review, we have highlighted the origin, development, subsets, and functions of pulmonary DCs, linking them to the activation of naïve T cells, effector T-cell formation, and immunity against fungal infections. Further, we have discussed in depth the DC-based vaccines used against several respiratory fungal pathogens viz. *Aspergillus* spp., *Cryptococcus* spp., *Coccidioides* spp., and *Paracoccidioides* spp. The utilization of various DC-based vaccine development strategies in animal models has made immense progress in the fungal field, further embracing a new perspective on the utilization of EV, nanoparticle-based, and mRNA-based vaccines that hold potential for the future of fungal vaccine development. Further elucidating the involvement, stability, longevity, plasticity, and dynamics of DC subsets in antifungal immunity will unveil novel avenues for the development of effective fungal vaccines.

## Data Availability

No new data were created or analyzed in this study.
